# Supplementary material for: Independent acoustic variation of the higher- and lower-frequency components of biphonic calls can facilitate call recognition and social affiliation in killer whales
Source: PLoS One. 2020 Jul 30;15(7):e0236749. doi: 10.1371/journal.pone.0236749 (PMC7392277; doi:10.1371/journal.pone.0236749)
Supplement: Attachment — Submitted filename: LineReview_PONE-D-20-05337.docx [file pone.0236749.s003.docx]

**Review of “Independent Cultural Change of Higher-Frequency component can facilitate call recognition in killer whales.”**

**Overall Review Summary**:

Overall, the study meets PLOSone’s Publication Requirements. I believe the study provides important insights into the potential functionality of biphonation in resident killer whale vocalizations and will be an important addition to the available literature on the subject – I recommend it be published, after the comments in this review are taken into account. The study is well executed. The field efforts to collect this acoustic data for the study for so many different family groupings is impressive and worthy of recognition. The author does an adequate job of citing and reviewing relevant literature on the subject from multiple taxa throughout the manuscript. The study was performed using established methods that have been published previously, with some modification. I would have liked to see this study include more than one population of resident killer whale to increase scope, such as also including recordings from the Northern Resident population, but this is not essential and the study still contributes in a meaningful way.

Overall, the manuscript is well written, but would benefit from copy-editing to address some issues in grammar and syntax. There are some sections that require re-wording for clarification and interpretability (see line-by-line comments below).

I may use the abbreviations ‘HFC’ and ‘LFC’ in some portions of my review, these just refer to ‘higher frequency component’ and ‘lower frequency component’, respectively.

**General comment:**

The author makes many references to ‘killer whales’, in general, throughout the manuscript, but the statements really only apply to a given ecotype (most often, ‘resident’ killer whales). And because the author only assesses vocalizations for resident-type killer whales in this study, the conclusions cannot be generalized over all killer whales/types due to the different acoustic behaviour, acoustic population sub-structuring, and vocalizations that other ecotypes exhibit. I suggest the author clarify their use of ‘killer whale’ in the paper to limit it to just the resident ecotype where appropriate.

The author also makes many references to ‘pod’ or ‘pods’ throughout the manuscript, but this can be misleading and ambiguous without providing a definition of the term to relate it to the previously published definitions of the term. See comments below for discussion on this.

**Line-referenced Comments**:

Title: I think the title can more accurately reflect the messages of the manuscript. First, it only speaks to the high frequency component, but the paper argues that the presence of both the high- and low-frequency component together provide killer whales with a tool that will ensure transmission of information in many more conditions/situations than either component individually. Second, the title only speaks to ‘call recognition’ but the paper’s main arguments revolve around the information that the calls carry – ie. Social group affiliation at various resolutions. Third, the title generalizes across killer whales, whereas I do not think these results can be interpolated beyond the ‘resident’ ecotype. I may recommend the following title (or something similar) to more accurately reflect the paper’s outcomes/arguments and reach: “Independent culturally-induced change of the higher- and lower-frequency components of biphonic calls can facilitate call recognition and social affiliation in resident killer whales”. This title more accurately represents the author’s conclusions in the discussion, and the fact that this really only relates to the resident killer whale ecotype (until similar research is conducted on the other ecotypes the use of the biphonation cannot be assumed to be the same without further study because different ecotypes seem to have different acoustic population sub-structuring, and thus the information communicated with the biphonation and the contexts it is used could be quite different).

Line 12: the author’s use of the term ‘pod’ needs to be defined. See further comments on this topic below.

Lines 28 and 29: Change “…component allows to recognize the family identity…” to “…component allows the recognition of family identity of a caller…”.

Line 40: The author uses the term ‘pod’ throughout the manuscript, however, the meaning of ‘pod’ is ambiguous. The author’s intended meaning of the term ‘pod’ needs to be defined. “Pod” has several meanings in the context of killer whales. “Pod” can be used in the traditional sense to describe a group of cetaceans (like a ‘herd’ of cattle, or ‘flock’ of birds). However, in killer whales, ‘pod’ was also defined by Bigg et al. 1990 as a functional social unit within the nested social structure of resident killer whales in British Columbia, as “A subpod made of groups who spend more than 50% of the time together“. Although this definition has been adopted by many researchers monitoring other killer whales around the world to describe social groupings, Ford and Ellis (2002, Reassessing the social organization of resident killer whales in British Columbia) determined that the ‘pods’ within Northern Resident Killer whales that were grouped base on the definition in Bigg 1990 no longer met this definition of ‘pod’ after ~30 years of monitoring the population (ie. a “pod” according to the social definition in Bigg et al 1990 is not a stable social grouping, and thus the use of the term ‘pod’ in this context should no longer be used). The author should define their intended meaning of ‘pod’ throughout the paper, or use a different descriptor. Or if the social groupings in their study population still adhere to Bigg’s definition of ‘pod’, then describe this (although, as mentioned, even though the population may still adhere to the definition, it likely won’t always, and a different term should be used).

Line 52: “New pods form gradually through the split of an ancestral pod after a matriarch dies…”. First, the use of ‘pod’ needs to be defined or not used (use ‘group’ instead), as described in comment above. Second, this is not the only mechanism that drives the creation of new groups in resident killer whales. New groups also form through group-splitting that takes place while the maternal ancestor is still alive (intra-geneological splitting), as described in Stredulinsky 2016: Determinants of Group Splitting in a Threatened Population of Fish-eating Killer Whales. Group splitting is a more complex process than this sentence suggests, and as such, so is dialect evolution.

Line 73-75: Strongly suggest a change in wording. Natal philopatry is not present in all types of killer whales (therefore the author should only relate this sentence to resident killer whales, not killer whales in general). Also, I would argue that family life is not ‘critical’ for survival in killer whales. Family cohesion has some very notable benefits that make maintaining family cohesion more advantageous than not in many killer whale societies, but it is not critical. There are some killer whale societies where emigration out of one’s family is important (such as in the West Coast Transient “Bigg’s” killer whale population). The author should refrain from generalizing across killer whales generally, and try to stick within the study population (resident killer whales).

Line 77: ‘Intentionally’ and ‘deliberately’ are synonyms.

Line 113-114: Were the hydrophones calibrated across frequencies? What method did the author use to determine the reportedly flat response (manufacturers documentation/technical specifications of the equipment? End-to-end system calibration including recording equipment? Hydrophone-only calibration?). If the hydrophones were not calibrated, this needs to be stated, with an indication as to how the flat response was determined.

Line 108-135: Were any observations about group behavioural state collected during the recordings? As noted in previous publications (e.g. Ford 1990), behavioural state can affect how whales produce vocalizations, with more aberrant versions of stereotyped calls being produced during periods of socializing. Since behaviour affects how calls are produced, it is possible that call similarity would be influenced by behavioural state. If some families included in the study were only recorded during bouts of intense socializing, the recorded calls may not accurately represent the structure of the stereotyped calls made by those families in other behavioural states more conducive to very stereotyped call production (such as foraging or consistent travel, etc). To truly compare similarity of call structure amongst family groups, shouldn’t behavioural state also be incorporated into the analysis as a variable?

Line 120: Did the author use a catalog to identify the individuals? Is the catalog citable? It should be cited if so.

Line 139-141: Why 10 calls per call type per group? Is 10 calls enough to capture the variability within the calls adequately? How were the 10 calls per type per group chosen to make up the dataset (were selection criteria based on quality or SNR (or other – like the first ten calls of each type in each recording of each family? The time-warping contour selection is too laborious for a larger sample size?))? Please state reasoning.

Did any examinations take place that looked at the overall quality of the final selected calls by family group? It seems to me that better quality calls may lead to better measurements in the dynamic time-warping analysis. If some groups had a higher proportion of poor quality calls, this may affect the comparisons.

The manuscript and interpretability of the results would be improved by including a few more details in the methods about the data set selection process.

Line 141: remove ‘each’ from ‘per each family’.

Line 142-146: Is the custom MATLAB contour extraction tool previously published? If so, please cite, or provide a few more details on the method: how did the operator determine they’ve selected enough points to adequately describe the contour? If the operator has to do this for each call, this may introduce subjectivity. Could this subjectivity bias the contour shape slightly of each call? If you repeated the point selection and extraction process 10 times from the exact same call clip would the resulting contours be the exactly the same? If not, how much variability exists among these contours?

Also, these methods need to be clearer on how the high-frequency and low-frequency contours were treated. Were they selected and extracted independently of each other (so there would be a HFC contour and a LFC contour for each call clip?)? In Line 168-169 the author states: “..and for the contours that consisted of both the components taken together.” Does this mean that the analyst selected points to extract the HFC and LFC as one contour containing features of both? More clarity on the contour extraction process and how the contours for both components were treated is needed to help interpretability and reproducibility.

Line 149-150: The author should briefly state why adopting a modified approach was necessary.

Line 150-155: This description needs to be clearer. It could be made clearer if supplemented by a figure of a contour and an example output from the time-warping algorithm to illustrate what they mean by “smaller frequency value by the larger frequency value at each point” and “a running tab was kept on the similarities of elements making up the curves while adding up these costs…”. More detail into how the cost matrix works would be great. A figure with a flow diagram of how the values from the time-warping get turned into percent similarity, then combined into the cost matrix resulting in a single value.

Line 158-160: This sentence is an exact replica of a sentence in the methods of Deecke and Janik 2006, citation [34] in this manuscript. This sentence must be re-worded to avoid plagiarism.

Line 161-162: the original ARTwarp algorithm was not developed by Deecke and Janik [34], but as Deecke and Janik [34] describe, it was originally developed by Carpenter and Grossberg (1987), and modified for use by Deecke and Janik.

Line 176-177: This part of the paper is very interesting and has the potential to provide great insight into how these two call components may be perceived and used by killer whales. However, using relative amplitude of the two components is only valid if the recording system’s frequency response is truly flat. The author previously mentions in the methods that the hydrophones used have a flat response, but provide no information for how that was determined. The detection range comparison compares the relative amplitude across frequencies, and thus, if the recording system does not actually have flat response, the relative contribution of the high and low frequency components will not reflect reality. For example, if the recording system is actually more sensitive in the 5-8kHz band than in the ~1kHz band, the amplitude of the high-frequency component would have been over-represented by assuming a flat response. The author should provide more information on how their recording system’s flat response was determined/verified (end-to-end calibration? hydrophone-only calibration? Manufacturers technical documentation? , etc).

Line 188: The author cites two papers as the source for the audiogram information used. However, the two papers present many different audiograms from many different individual killer whales (and even the composite audiograms presented in the two papers are slightly different). For reproducibility, the author must state which specific audiogram was used.

Line 217-221: Figure 2: Figure 2’s axes need to be labelled. Only the y-axis is labelled. Also, the x-axis label has a spelling error.

Line 242: “All these calls…” This should be more specific, such as: “All of the calls without detectable high-frequency components…”

Line 271: Discrimination of a *caller’s orientation*, not ‘direction’. The direction to a caller is not related to the high-frequency component’s directionality (their hearing is directional and thus can infer direction to a caller inherently). The high-frequency component gives information about the *orientation* of the caller relative to the receiver.

Line 276-278: Direction of movement does not necessarily equate to the direction of calling. The whale could temporarily face a certain direction to emit a call, then continue on it’s intended path. Also, the high-frequency component is not necessary for determining direction of travel. The caller only needs to emit two omnidirectional sounds of any type while travelling and the listener can discern direction of travel. In this context, the higher frequency component’s directionality can only provide the caller’s orientation. And the orientation information is quite ambiguous, especially considering the 4D environment the whales live in and the number of possible orientations that are possible for the same amplitude ratio of the high to low frequency component. And on top of this, the whales make other types of directional sounds, so the HFC isn’t the only vocalization that can elude to orientation of the caller (I think echolocation is likely a better indication of orientation because it is made very frequently and consistently and the returning echoes from features can also be used…).

This is all to say that the author can strengthen their argument that the HFC is not solely used for orientation/group coordination using the above points. Also, the discussion can be improved by speaking to the other potential reasons for the directionality in the high-frequency component. The directionality of the HFC can be more useful than for just providing orientation information: the directionality of the component would allow a caller to emit family/clan membership information towards specific whales or groups within an aggregation selectively (just as a human in a noisy room would yell (everyone in the room would hear), but ALSO make eye contact (directional component) with a person on the other side of the room if they were the intended target of such communication). The directionality can also help increase the SNR in the direction of a receiver in noisy conditions to be heard better in noise – not just the simple presence of the high frequency component, because that can be masked quite easily in some situations, but the ability to create a higher SNR by directing the call towards other members of the group in noisy conditions).

I think the directionality aspect of the HFC is a very important functionality of the HFC and should be discussed more in the discussion (because it ultimately helps out the author’s arguments and final conclusions). Being able to provide family membership more reliably is fine, but the ability to provide family membership in certain directions is a very useful tool for a very social species (including in noisy conditions).

Line 282-284: I don’t think this is necessarily true. The structure of a tone (whether it is frequency modulated or not) can also affect its detectability in different noise conditions. There are types of background noise where a non-frequency modulated tone would be more detectable than a modulated one, but there are also situations where a modulated tone would be more detectable than a non-modulated one. This argument needs to be strengthened.

Line 290-292: I think this sentence seems a little abrupt, as these uses of biphonation haven't been brought up in the paper before, and also not in relation to killer whales - the abruptness hides the intended purpose of the sentence. I suggest changing the sentence to make it less abrupt, and capture the attempted purpose of the sentence: "Additional hypotheses for the function of biphonation in other species have included: increasing unpredictability and providing an indication of physical condition [19]. However, these are likely not appropriate considerations as possible functions of biphonation in killer whales. These hypotheses suggest that the caller can choose when to include or not include a biphonic component in each call it makes. However, calls that comprise killer whale dialects are stereotyped and ..." Or something like that.

Line 294: The use of the term ‘mandatory’ is slightly misleading. Yes, in the specific call types that contain HFC’s, the HFC component is mandatory (the whale does not choose to whether include or remove the component), but the way the sentence is written it could be interpreted that the HFC is mandatory in *any* call the whale makes.

Line 297: “probably on the family level…” Why only at the family level? In this manuscipt, the author only examined the frequency-time component of the call components, but more information is provided in a vocalization, such as it’s timbre? Could this not carry individual information in the HFC/LFC? There needs to be a citation for why the author believes only family level is possible in killer whales – there is arguably strong pressure for individual recognition in resident killer whales – such as in kin-directed prey-sharing, Wright et al. 2017.

Line 312: ‘shifted’ may not be the appropriate word. “concentrated” or “is more pronounced in lower frequencies”, etc. The noise isn’t shifted to lower frequencies, it is just the nature of most anthropogenic ambient noise.

Line 319-320: The author should also consider addressing this argument in relation to Viers et al 2016: “Ship noise extends to frequencies used for echolocation by endangered killer whales”. It should be noted that, at times, even the HFC can be masked by ship/anthropogenic noise. But the author can also add another advantage of the directionality of the HFC in this context. If noise levels are high, even to the point of starting to mask the HFC, the caller can point in the direction of the receiver while calling to increase the HFC’s SNR. This all adds to the potential function of this component.

Line 324-325: “…better hearing sensitivity of killer whales to higher frequencies.” Cite [35, 36] here.

Line 338: This is a very cool possibility, but I wonder if it is meaningful biologically? The heterodyne frequencies only show up when call is good quality (high SNR or close), and in this sense, it may not be that effective when in noise because to resolve it the whales need to be relative close to one another, and if they are relatively close, they will already be able to discern the low frequency component?

Line 357: “pods” should be replaced by “groups” or “social units” for clarity.

Line 362: “This process of cultural change is called cultural evolution.” The author is not defining what cultural evolution is for the first time in this paper. Please cite.

Line 363: use of ‘pods’ needs to be defined or changed to ‘groups’.

Line 368: “…probability that both components would converge in two pods is very low.” The probability is likely relative to whether the two ‘pods’ are members of the same or different populations. And if in part of the same population, relative to the overall population size and the frequency that different groups in that population encounter one another.

Line 371-377: This paragraph would benefit from re-wording to provide better clarity.

Line 402-405: I think these conclusions can benefit from a more thorough discussion of the advantages of directionality (as mentioned previously in these comments).

**Supplemental Information**:

S1: I noticed that the matrices for the K5 and K7 call types are different sizes. Why are the K5 datasets 150x150 whereas the K7 datasets are 140x140? In the methods the author describes data collection as: 14 families, 10 calls of each type per family, so shouldn’t they both be 140x140? I may have misunderstood something? If not, then the author should address this in the methods.
