# Supplementary material for: Independent acoustic variation of the higher- and lower-frequency components of biphonic calls can facilitate call recognition and social affiliation in killer whales
Source: PLoS One. 2020 Jul 30;15(7):e0236749. doi: 10.1371/journal.pone.0236749 (PMC7392277; doi:10.1371/journal.pone.0236749)
Supplement: Attachment — Submitted filename: Response to Reviewers.docx [file pone.0236749.s004.docx]

Dear Editor and Reviewer #4,

I have addressed all the comments in the revised version of the manuscript.

Reviewer #4: I have a few minor comments that could improve the manuscript further.

Ln 26 – could change ‘perceived amplitude’ to ‘sensation level’ to use standard terminology

Response: Changed.

Ln 273 – this section has ‘over distance’ in the title, but no consideration of propagation effects or distance over which they might be used are mentioned here. Consider to change the title of this section. Alternatively, you could mention that frequencies of the HFC will absorb roughly 1dB/km more than the LFC and consider the consequences of this difference on the typical detection ranges indicated in Miller et al., 2006 – At 10km distance, the HFC will have lost 10dB more than the LFC.

Response: I have added the explanation about the frequency-dependent absorption and the reference to Miller (2006).

Ln 279 – the HFC may also not be detectable if the whales were oriented away

Response: If the whale is close, the HFC will be detectable even if it is oriented away (I have seen it many times when whales were passing our boat, calling first towards, and then away from the hydrophone – the HFC was weaker in the latter case, but still detectable). Therefore, the orientation away decreases the received level of the HFC, but it becomes undetectable due to transmission loss, which is already mentioned in the text.

Ln 338: could change ‘mandatory’ to ‘obligatory’

Response: Changed.

Ln 457: change ‘the main’ to ‘a likely’ – until we are able to test these different ideas using playback experiments all of the proposed functions remain somewhat speculative.

Response: Changed.

Ln 461: the directionality means that this will not be audible as far to receivers that aren’t ahead of the signaller. This is a potential drawback to its use as a general family indicator, so it may not really be ‘especially suitable’. The combination of both components does provide a redundancy of information that is beneficial for these animals to maintain contact with preferred group members.

Response: I have deleted the statement that the directionality makes these calls ‘especially suitable’ and added the statement that combination of both components provides a redundancy that is beneficial in their environment.
